# Identification of Survival Risk and Immune-Related Characteristics of Kidney Renal Clear Cell Carcinoma

**DOI:** 10.1155/2022/6149369

**Published:** 2022-07-04

**Authors:** Xiaobin Wu, Yonghui Liang, Xian Chen, Xiangyang Long, Wujun Xu, Li Liu, Binhui Wang, Xiong Zou

**Affiliations:** ^1^The Second Affiliated Hospital, Department of Urology, Hengyang Medical School, University of South China, Hengyang, Hunan 421001, China; ^2^Department of Nursing, Guangxi Meiao Maternity Hospital, Nanning, Guangxi 530021, China; ^3^Department of Urology, The First Affiliated Hospital of Guangxi Medical University, Nanning, Guangxi 530021, China; ^4^Center for Genomic and Personalized Medicine, Guangxi Medical University, Nanning, 530021 Guangxi, China; ^5^Guangxi Collaborative Innovation Center for Genomic and Personalized Medicine, Nanning, Guangxi 530021, China; ^6^Guangxi Key Laboratory for Genomic and Personalized Medicine, Guangxi Key Laboratory of Colleges and Universities, Nanning, Guangxi 530021, China

## Abstract

**Background:**

Immunity exerts momentous functions in the progression and treatment of kidney renal clear cell carcinoma (KIRC). A better understanding of the relationship between KIRC and immunity may make a great contribution to evaluating the prognosis and immune-related therapeutic response of KIRC.

**Methods:**

A series of information such as RNA sequence, clinical data, and tumor mutation burden (TMB) of KIRC patients were downloaded through The Cancer Genome Atlas (TCGA). Next, combining the survival information and gene expression data of TCGA and Gene Expression Omnibus (GEO), we established an immune gene-related prognosis model (IGRPM) and analyzed it. Then we constructed a nomogram which was convenient for clinicians to judge the prognosis of KIRC. Last but not the least, the expressions of some genes used to construct IGRPM in early KIRC, and adjacent normal tissues were verified through real-time fluorescence quantitative polymerase chain reaction (RT-qPCR). Perl (strawberry-perl-5.30.0.1-64bit), R software (4.0.3), and GraphPad Prism 7 were used to process the relevant data.

**Results:**

The single-sample gene set enrichment analysis (ssGSEA) showed that there were significant differences in StromalScore, ImmuneScore, ESTIMATEScore, TumorPurity, 22 kinds of human immune cells infiltration, and HLA genes expression between high immunity group (Immunity_H) and low immunity group (Immunity_L). The Immunity_H expressed more immune-related genes and enriched more immune-related functions than the Immunity_L. In addition, compared with the low-risk group, the high-risk group had worse survival outcome and higher TMB. Combining IGRPM-based risk characteristic and TMB, we found that low-TMB + low-risk was the most beneficial to the survival outcome of KIRC patients. The risk characteristic based on IGRPM could be used as an independent prognostic factor for KIRC, and the nomogram constructed for evaluating the prognosis of KIRC showed excellent predictive potential. The RT-qPCR results suggested that not all the genes used to construct IGRPM showed differential expression in early KIRC compared with adjacent normal tissues, but all these genes had significant influence on the prognosis of KIRC.

**Conclusion:**

These comprehensive immune assessments and survival predictions, integrating multiple aspects of data and clinical information, can provide additional value to the current Tumor Node Metastasis staging system for risk stratification of KIRC and may facilitate the development of KIRC immunotherapy.

## 1. Introduction

Renal cell carcinoma (RCC), which originates from nephron [1], is one of the 10 most common malignancies in humans [2]. However, kidney renal clear cell carcinoma (KIRC) is one of the most invasive pathological types of RCC and the main pathological type [3], accounting for about 75% of all RCC [4]. Moreover, KIRC is a crucial cause of cancer-related death worldwide [5]. It is important to analyze the clinicopathological features of KIRC patients according to the WHO classification and Tumor Node Metastasis (TNM) staging for selecting appropriate treatment methods [6]. However, there may be significant differences in prognosis even in patients with the same TNM staging and pathological grade after similar treatment [7, 8], indicating that KIRC is a heterogeneous tumor, and the existing clinicopathological features and classification are not sufficient to evaluate the prognosis and risk stratification of KIRC patients. Therefore, finding a new method with high predictive value is of great significance to improve the prognosis of KIRC.

The development and occurrence of tumor and the effectiveness of immunotherapy are closely related to tumor immune microenvironment (TIM) [9, 10]. More and more studies have emphasized that there is heterogeneity in the proportion of immune-related genes and immune cell populations in tumors [11, 12], which may be one of the key reasons for different prognosis of patients. Oncologists are committed to exploring the immune responses and its regulatory mechanisms and screening immune-related biomarkers in order to coordinate the immune microenvironment of tumors and improve survival prognosis [13]. Immune cells are related to tumor metastasis and invasion and are potentially important forces to slow down or inhibit tumor growth [14]. However, although the research on the relationship between KIRC and immunity has increased in recent years, there is still a lack of research that can accurately predict the survival of KIRC patients in terms of immunity.

With the sharing of massive data, bioinformatics analysis has been widely used in the study of tumor prognosis [15]. In this study, we constructed an immune gene-related prognosis model (IGRPM) for patients with KIRC using data from The Cancer Genome Atlas (TCGA) and Gene Expression Omnibus (GEO). In addition, we systematically understood and distinguished the immune-related characteristics of KIRC, and looked into the correlation of tumor immune-related genes with TIM in depth. Additionally, we constructed a nomogram which was convenient for clinical application and verified its similarity with the ideal model. Finally, we verified the expressions of some of these genes used to construct IGRPM in early KIRC and adjacent normal tissues through real-time fluorescence quantitative polymerase chain reaction (RT-qPCR). Moreover, we confirmed that all the genes used to construct IGRPM had a significant effect on the prognosis of KIRC through UALCAN database.

## 2. Materials and Methods

### 2.1. Data Source

We downloaded transcriptome and clinical data of KIRC from TCGA (https://portal.gdc.cancer.gov/; December, 2021) and GEO (https://www.ncbi.nlm.nih.gov/geo/; December, 2021; GSE29609). The transcriptome data (HTseq-FPKM) of TCGA included 539 tumor samples and 72 paracancerous normal samples. 537 KIRC samples were downloaded from TCGA including clinical data. Masked somatic mutation data for 339 KIRC samples were downloaded from TCGA, of which tumor mutation burden (TMB) was successfully calculated for 336 samples. The GSE29609 cohort including 39 KIRC samples from GEO database and 530 KIRC samples from TCGA were used to construct prognostic models. During the data processing, we deleted some samples with incomplete information. The genetic data of related samples were converted into matrix files by Perl software (strawberry-perl-5.30.0.1-64bit). We analyzed the data through R software (4.0.3).

### 2.2. Single-Sample Gene Set Enrichment Analysis (ssGSEA) and Immune Classification Analysis

Using the ssGSEA algorithm of R software package (“GSEABase,” “limma,” and “GSVA”), the immune-related characteristics of each sample in TCGA-KIRC were evaluated comprehensively, based on 29 immune gene sets, including different immune cells, immune-related functions, and immune-related genes [16]. According to the immunological characteristics of the samples, 539 tumor samples from TCGA were analyzed by Ward's linkage and Euclidean distance, which were divided into high immunity group (Immunity_H) and low immunity group (Immunity_L) [17]. The accuracy of immunotyping of KIRC patients was verified by R package (“Rtsne”).

### 2.3. Estimation of Tumor Purity and Immune Characteristics

We estimated the infiltration of stromal cells and immune cells in 539 KIRC tumor samples from TCGA by R software package (“estimate”), thus determining the StromalScore, ImmuneScore, ESTIMATEScore, and TumorPurity of the samples [18]. We also calculated the abundance of 22 types of human immune cells in each sample by CIBERSORT method [19] and compared the cell composition of the two subtypes in 539 KIRC samples of TCGA.

### 2.4. Differentially Expressed Immune Genes in Immunity_H and Immunity_L

We identified the differentially expressed genes (DEGs) between Immunity_H and Immunity_L by “limma” package [20]. Then, the DEGs were intersected with the immunologically relevant list of genes downloaded from ImmPort to obtain the differentially expressed immune genes (DEIGs) [21]. In addition, we also compared the differences in the expressions of HLA-related genes in Immunity_H and Immunity_L.

### 2.5. Functional Enrichment Analysis

We performed gene set enrichment analysis of 539 KIRC patients from TCGA by GSEA (Version4.1.0), in order to reveal the key signal pathways and functions involved in the two subtypes [22]. The screening thresholds of KEGG pathway and GO analysis in the Immunity_H and Immunity_L were *p* < 0.05. The enriched pathways were visualized with R packages (“reshape” and “ggplot2”).

### 2.6. Analyses of Prognosis Associated Immune Genes and Regulatory Networks

We obtained the DEIGs shared by TCGA and GEO through R packages (“limma” and “sva”). Next, we merged the shared DEIGs with survival data from TCGA and GEO into a single file and carried out univariate Cox proportional hazard regression analysis with R package (“survival”). It was considered that the survival risk gene of *p* < 0.001 was the prognosis associated immune genes (PIGs) of KIRC patients. Next, we used the CISTROME (http://www.cistrome.org/) to find transcription factors (TFs) correlated to cancer occurrence and progression [23, 24], retrieved the differentially expressed transcription factors (DETFs) from DEGs, and constructed the regulatory network of DETFs and PIGs by Pearson's correlation coefficient analysis. Correlation coefficient (|cor|) >0.4 and false discovery rate (FDR) <0.001 were the cut-off point of significant correlation. For coexpressed PIGs and DETFs, protein-protein interaction (PPI) analysis was also performed using STRING (https://cn.string-db.org/).

### 2.7. Construction of Immune Gene-Related Prognostic Model Based on TCGA and GEO Data

Using the LASSO Cox regression model based on R packages (“Matrix,” “glmnet,” and “survival”) and taking PIGs as the research object, an optimal immune gene-related prognosis model (IGRPM) was constructed for KIRC. The formula for calculating the risk score is as follows: the risk score = *Σ*_*k*_^*n*^Expression_(*k*)_ × coef_(*k*)_. In this formula, Expression_(*k*)_ denotes the expression level of gene *k* in the TCGA and GEO samples, and coef_(*k*)_ denotes the regression coefficient of characteristic gene *k*. All patients were separated into two groups based on IGRPM's median risk score: low-risk and high-risk; and R packages (“survival” and “survminer”) were used to conduct the survival analysis. In order to reflect the predictive ability of risk characteristic based on IGRPM, we created a receiver operating characteristic curve (ROC) depending time through R packages (“survminer,” “survival,” “rms,” and “timeROC”) and calculated the area under the curve (AUC) of different survival times. Spearman correlation analysis of R software was used to explore the correlations of IGRPM-based risk characteristic with immune cells infiltration and TMB. Finally, we performed multivariate and univariate Cox regression analyses of IGRPM-based risk characteristic as well as other clinicopathological parameters to judge if IGRPM-based risk characteristic could be applied as an individual prognostic indicator for KIRC patients. Last but not the least, we constructed a nomogram through the risk characteristic based on IGRPM and the other clinicopathological features; what is more, the proposed nomogram was tested using ROC and calibration curve analyses to see if it was suitable for clinical application.

### 2.8. Real-Time Fluorescence Quantitative Polymerase Chain Reaction and UALCAN

Three early KIRC samples and adjacent normal tissues were collected from the Second Affiliated Hospital of University of South China. The clinicopathological characteristics of the patients were shown in Supplementary table 1. Total RNA was extracted by the FastPure Cell/Tissue Total RNA Isolation Kit V2 (RC112-01, Vazyme). PrimeScript RT reagent kit (RR037A, Takara) and Bio-Bad T100 Thermal Cycler according to the manufacturer's instructions were used to perform reverse transcription to synthesize cDNAs. Reverse transcription was performed with a final quality of 1000 ng of RNA. For quantification of gene expression levels, we performed RT-qPCR using FastStart Universal SYBR Green Master (04913914001, Roche) and Light Cycler96 Instrument (Roche). The comparative cycle threshold (Ct) method was used for relative quantification of each target [25–27]. △Ct = Ct (gene of interest) − Ct (GAPDH). The GAPDH was used as an internal reference. The 2^-*ΔΔ*Ct^ method was applied to calculate the expression of target genes in tumor tissues compared with adjacent normal tissues. All groups set up three biological replicates, and experiments were repeated three times for each sample. Meanwhile, we explored the effect of all genes used to construct IGRPM on the overall survival (OS) of KIRC through the UALCAN database. The following primers were used for RT-qPCR.

GAPDH:

5′-ACAACTTTGGTATCGTGGAAGG-3′(Forward),

5′-GCCATCACGCCACAGTTTC-3′(Reverse);

OASL (2′-5′-Oligoadenylate Synthetase Like):

5′-CTCCCACACTCACATCTATCTG-3′(Forward),

5′-ACTGTCTTGGATGCCATAGATC-3′(Reverse);

THRB (Thyroid Hormone Receptor Beta):

5′-CTCATCAAAACTGTCACCGAAG-3′(Forward),

5′-CAACTTTTTGGCAAAATCCACC-3′(Reverse);

CLDN4 (Claudin 4):

5′-CCACAACATCATCCAAGACTTC-3′(Forward),

5′-CAGAATACTTGGCGGAGTAAGG-3′(Reverse);

SAA1 (Serum Amyloid A1):

5′-CAGACAAATACTTCCATGCTCG-3′(Forward),

5′-TCTCTGGATATTCTCTCTGGCA-3′(Reverse);

NR3C2 (Nuclear Receptor Subfamily 3 Group C Member 2):

5′-GCTGGAAGAAATGATTGCATCA-3′(Forward),

5′-AACTTCTTTGACTTTCGTGCTC-3′(Reverse);

LGR4 (Leucine-Rich Repeat-Containing G Protein-Coupled Receptor 4):

5′-CACACTTGGGCCAATAACTAAC-3′(Forward);

5′-ACAAAGTCTTTTGCTGCTAAGG-3′(Reverse).

### 2.9. Statistical Analysis

The results of RT-qPCR were statistically analyzed by *T*-test using GraphPad Prism 7. *p* < 0.05 indicated statistically significant difference.

## 3. Results

### 3.1. Identification of Two Subtypes of KIRC By Immune Gene-Related Characteristics of Samples

**Figure 1:**
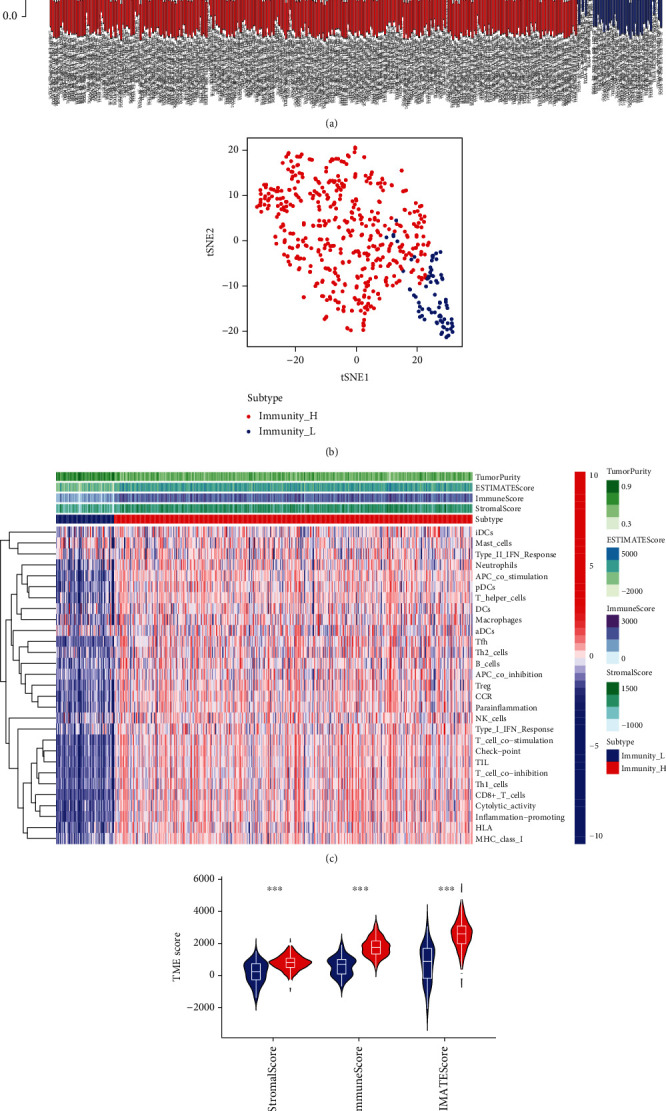
Immune stratification and clustering in patients with KIRC. (a) According to ssGSEA analysis, KIRC was divided into Immunity_H and Immunity_L based on immune score and hierarchical clustering algorithm. (b) The cluster analysis of KIRC patients was further confirmed by tSNE. (c) The immune characteristics of TCGA-KIRC cohort and the landscape of tumor microenvironment. (d) ImmuneScore, StromalScore, and ESTIMATEScore were compared in Immunity_H and Immunity_L. ∗*p* < 0.05, ∗∗*p* < 0.01, and ∗∗∗*p* < 0.001.

To systematically characterize the immunological features of KIRC, the ssGSEA algorithm was used to evaluate 539 tumor samples from the TCGA-KIRC cohort through 29 immune cells or immune-related functions. According to ssGSEA analysis, the immune score and hierarchical clustering algorithm were performed for each sample, and the 539 tumor samples of TCGA were divided into two clusters: Immunity_L and Immunity_H (Figure 1(a)). Moreover, we further confirmed the cluster analysis of KIRC patients by using tSNE algorithm and obtained similar results (Figure 1(b)). In addition, we visually showed the scores of 29 immune cells or immune-related functions as well as immune microenvironment scores in Immunity_H and Immunity_L through heat map and violin plot (Figures 1(c) and 1(d)). According to the two pictures, it can be concluded that the StromalScore, ImmuneScore, and ESTIMATEScore in Immunity_H were higher than those in Immunity_L, while the Immunity_H's TumorPurity was lower than the Immunity_L's TumorPurity (wilcox.test).

### 3.2. Comparison of Immune-Related Characteristics in the Two Subtypes

**Figure 2:**
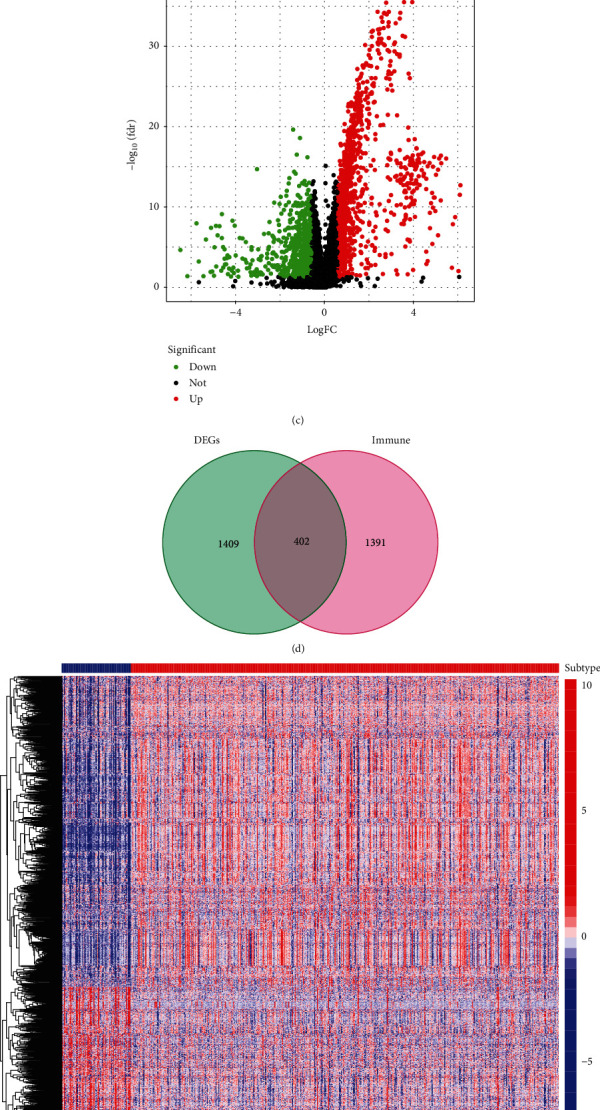
The levels of immune cells infiltration, HLA genes expression, DEGs, and DEIGs analyses in Immunity_H and Immunity_L. (a) Comparison of the levels of immune cells infiltration between Immunity_H and Immunity_L. (b) Comparison of HLA genes expression levels between Immunity_H and Immunity_L. (c) Volcano plot of all DEGs, Immunity_H vs. Immunity_L, |LogFC| >0.585, and FDR <0.05. (d) Venn diagram of DEGs and immune genes. (e) Landscape of DEGs between Immunity_H vs. Immunity_L. (f) Landscape of DEIGs between Immunity_H and Immunity_L. ∗*p* < 0.05, ∗∗*p* < 0.01, and ∗∗∗*p* < 0.001.

CIBERSORT algorithm was used to compare the infiltration degree of 22 kinds of human immune cells in Immunity_H and Immunity_L. We discovered that the fractions of memory B cells, gamma delta T cells, follicular helper T cells, CD8 + T cells, regulatory T cells (Tregs), activated memory CD4 + T cells, resting memory CD4 + T cells, resting dendritic cells, and M1 macrophages in Immunity_H were significantly higher than that in Immunity_L; however, the fractions of resting Mast cells, M2 Macrophages, and M0 Macrophages in Immunity_H were significantly lower than that in Immunity_L (wilcox.test) (Figure 2(a)). Next, we also explored the expressions of HLA-related genes between Immunity_H and Immunity_L. To our surprise, the expressions of all HLA-related genes in Immunity_H were significantly higher than that in Immunity_L (Figure 2(b)). All these results indicated that it was very reasonable and of great significance for us to classify KIRC patients into the Immunity_H and Immunity_L.

Furthermore, we analyzed the DEGs between subtypes and obtained the DEIGs based on ImmPort database. Compared with Immunity_L, Immunity_H had more DEGs upregulation (Figure 2(c)). 1811 genes in the two subtypes were authenticated as DEGs, of which 402 were DEIGs (Figures 2(d)–2(f)).

### 3.3. The KEGG and GO Analyses of Two Subtypes

**Figure 3:**
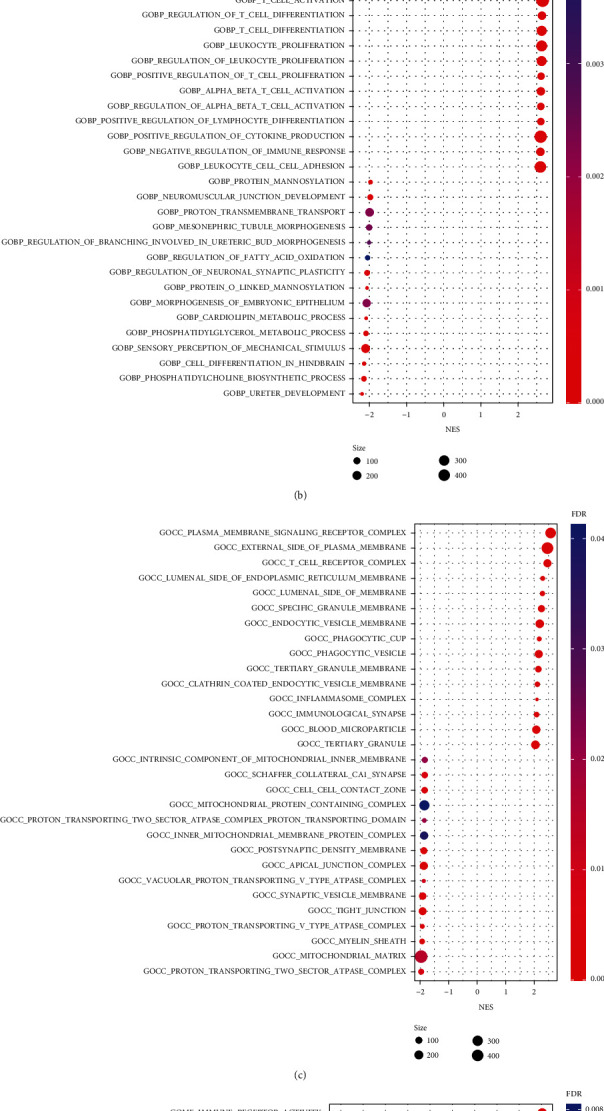
The GO and KEGG pathway analyses of Immunity_H and Immunity_L in KIRC. (a) Biological processes involved in Immunity_H and Immunity_L. (b) Cellular components involved in Immunity_H and Immunity_L. (c) Molecular functions involved in Immunity_H and Immunity_L. (d) KEGG pathways involved in Immunity_H and Immunity_L.

We carried out KEGG and GO analyses according to DEGs in order to further understand the functional differences between Immunity_H and Immunity_L (Immunity_H vs. Immunity_L). We found that the KEGG pathways enriched in Immunity_H were mainly antigen processing and presentation, cytokine-cytokine receptor interaction, T cell receptor signaling pathway, natural killer cell-mediated cytotoxicity, JAK-STAT signaling pathway, cell adhesion molecules cams, toll-like receptor signaling pathway, chemokine signaling pathway, B cell receptor signaling pathway, NOD-like receptor signaling pathway, complement and coagulation cascades, and leukocyte transendothelial migration; all these pathways are closely related to immunity (Figure 3(a)). However, there was no significant correlation of the enriched pathways with immunity in Immunity_L (Figure 3(a)). Biological process (BP), cellular component (CC), and molecular function (MF) of GO analysis also showed similar results as KEGG (Figures 3(b)–3(d)). These results demonstrated that the immunotyping of KIRC was accurate and that the immune activity was significantly improved in the Immunity_H.

### 3.4. Screening of PIGs and Construction of Regulatory Networks

**Figure 4:**
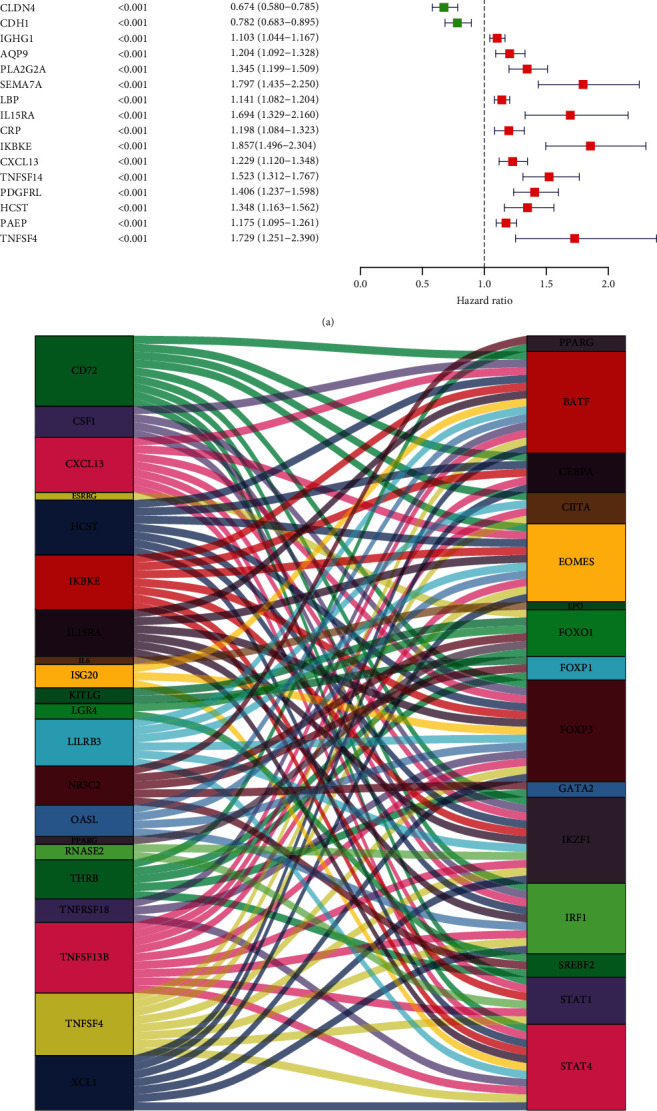
Analyses of prognostic-related genes and their regulatory networks and interactions with transcription factors in KIRC. (a) Forest plot based on univariable Cox proportional hazards regression analysis showing the PIGs and their hazard ratios. (b) Alluvial diagram of the PIGs and DETFs revealing their regulatory network. (c) PPI network between PIGs and DETFs.

Next, combining the survival and gene expression data of TCGA and GEO, we identified 47 PIGs and calculated the hazard ratio (HR) by univariate Cox proportional hazard regression analysis (Figure 4(a)). There were 35 high-risk PIGs (HR >1) and 12 low-risk PIGs (HR <1). To better understand the multidimensional and complex regulatory network of PIGs involved in the onset and progression of KIRC, we first investigated the coexpression analysis of PIGs and DETFs. Surprisingly, all PIGs and DETFs that met our filter conditions showed a positive correlation (Supplementary table 2). The regulatory network between PIGs and DETFs was shown in Figure 4(b). In order to further confirm that there were significant correlations of DETFs with PIGs, we used STRING to analyze PPI and observed that there were complicated connections within them (Figure 4(c)).

### 3.5. Combining TCGA and GEO Samples to Construct an IGRPM of KIRC through Lasso Cox Regression Analysis

**Figure 5:**
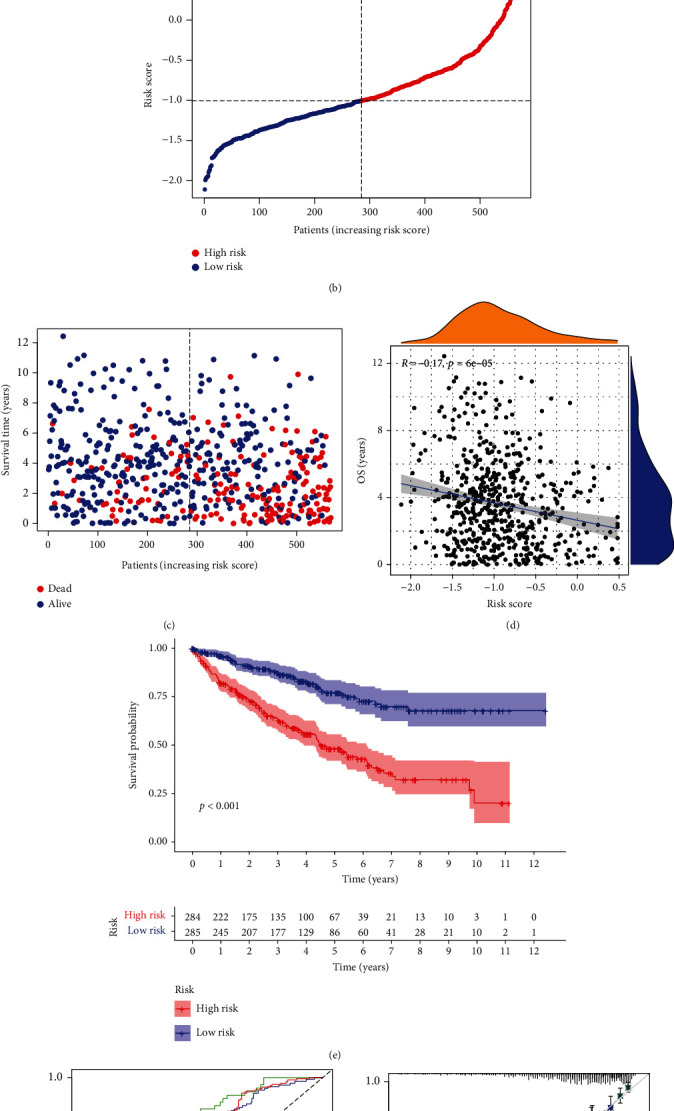
Construction and analysis of IGRPM. (a) Cross-validation for tuning parameter selection in the lasso model. (b) Risk score distribution of KIRC patients in the combined TCGA and GEO cohorts. (c) Distribution of the survival time and survival status in the combined TCGA and GEO cohorts. (d) Correlation analysis of OS and risk score in the combined TCGA and GEO cohorts. (e) Kaplan-Meier survival analysis for different risk groups in the combined TCGA and GEO cohorts. (f) The 1-, 3-, and 5-year ROC and AUC predicted based on the risk characteristic of IGRPM in the combined TCGA and GEO cohorts. (g) The calibration diagram of IGRPM-based risk characteristic for the combined TCGA and GEO cohorts. OS: overall survival.

To better utilize of the PIGs of KIRC, the dimension of PIGs was reduced by lasso Cox regression analysis. Combining with the KIRC cohorts of TCGA and GEO, we constructed an IGRPM containing 17 key genes to predict OS (Figure 5(a)). The genes and coef used to determine the risk score for each subject were shown in Supplementary table 3. The corrected gene expression level, survival time, and survival status of TCGA and GEO samples were shown in Supplementary file1. We evaluated the risk score for KIRC patients of GSE29609 and TCGA and separated them into high-risk and low-risk groups based on the median risk score (Figure 5(b)). It is not difficult to find that the high-risk group had considerably more deaths than the low-risk group (Figure 5(c)). In addition, our further analysis showed that the risk score was negatively correlated with OS (Figure 5(d)), suggesting that the OS of KIRC patients gradually decreased with the elevation of the risk score. We also drew the Kaplan-Meier curve of KIRC patients with R packages (“survival” and “survminer”). The results demonstrated that patients in the high-risk group had a significantly shorter survival time than those in the low-risk group (*p* < 0.001, Figure 5(e)). What is more, we conducted a time-dependent ROC analysis to better examine the prediction accuracy of IGRPM-based risk characteristic, and the predicted AUC at 1, 3, and 5 years was 0.751, 0.717, and 0.728, respectively (Figure 5(f)). Additionally, the calibration diagram revealed the anticipated value of IGRPM-based risk characteristic was basically consistent with the real value (Figure 5(g)).

### 3.6. The Correlations between IGRPM-Based Risk Characteristic and Immune Cells Infiltration and TMB

**Figure 6:**
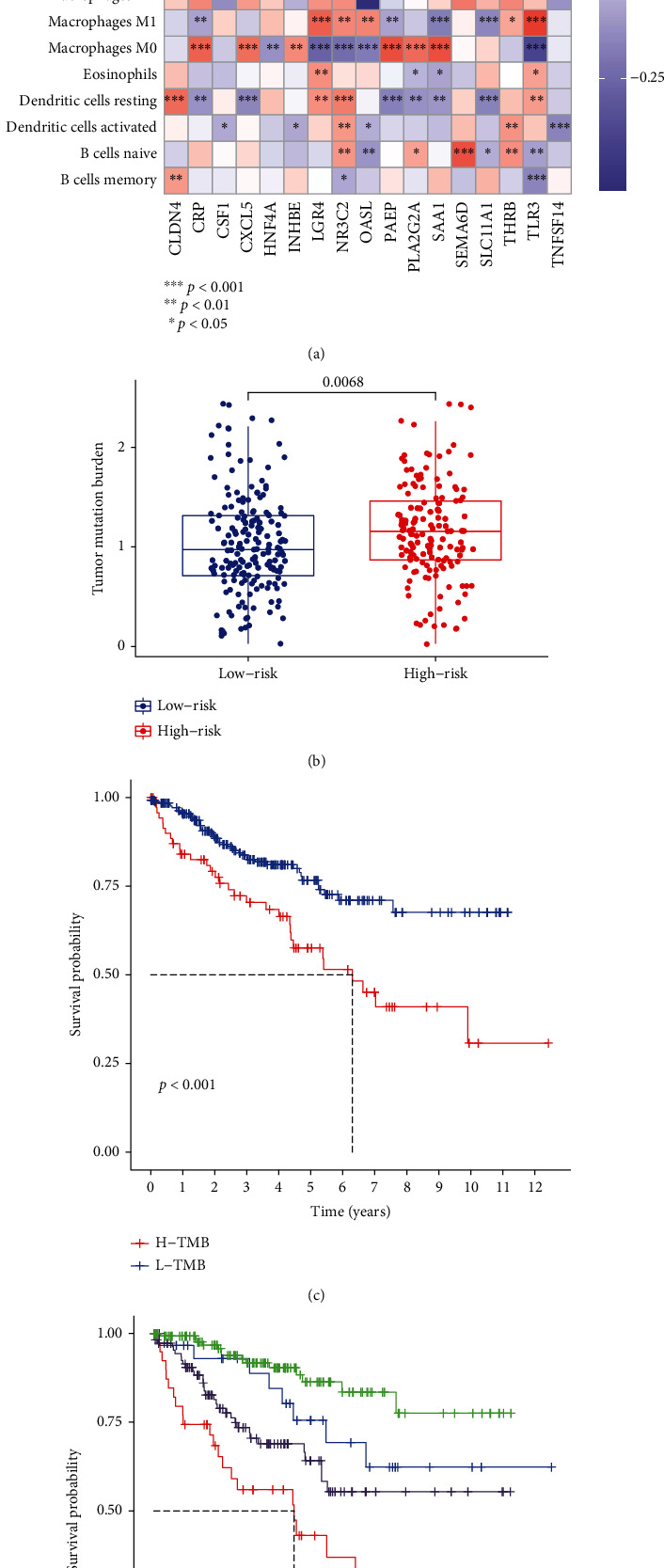
The correlations between IGRPM-based risk characteristic and immune cells infiltration and TMB. (a) Heat map showing correlation of 17 genes used to construct IGRPM with 22 kinds of human immune cells. (b) Comparison of TMB between the low-risk and high-risk groups in TCGA-KIRC cohort. (c) Kaplan-Meier survival analysis based on the TMB in the TCGA-KIRC cohort. (d) Combining with the risk characteristic based on IGRPM and the TMB in the TCGA-KIRC cohort, the survival analysis of the four groups stratified was carried out.

Due to the absence of some data from GEO, we performed further analysis based on TCGA data. We analyzed the correlations between 17 genes used to construct IGRPM and 22 kinds of human immune cells by “spearman” method. Our results revealed that 17 key genes were closely associated with a variety of immune cells (Figure 6(a)), which once again demonstrated the importance of the immune microenvironment in the prognosis of KIRC patients. We further explored whether TMB differed in high-risk and low-risk groups. The results demonstrated that the TMB of high-risk group was substantially elevated compared with that of low-risk group (Figure 6(b)). Moreover, the survival time of patients with high TMB (H-TMB) was significantly shorter than that of patients with low TMB (L-TMB) (Figure 6(c)). Considering that both TMB and IGRPM had significant effects on the survival of patients with KIRC, we further investigated if the combination of IGRPM and TMB could better predict the survival outcome of KIRC patients. According to the risk score and TMB, we separated all samples from TCGA into 4 groups, including L-TMB + high-risk group, L-TMB + high-risk group, H-TMB + high-risk group, and H-TMB + low-risk group. The result revealed that the survival outcome of KIRC patients with L-TMB + low-risk group was the best in four groups (Figure 6(d)).

### 3.7. Independent Prognostic Analysis and Establishment and Verification of Nomogram

**Figure 7:**
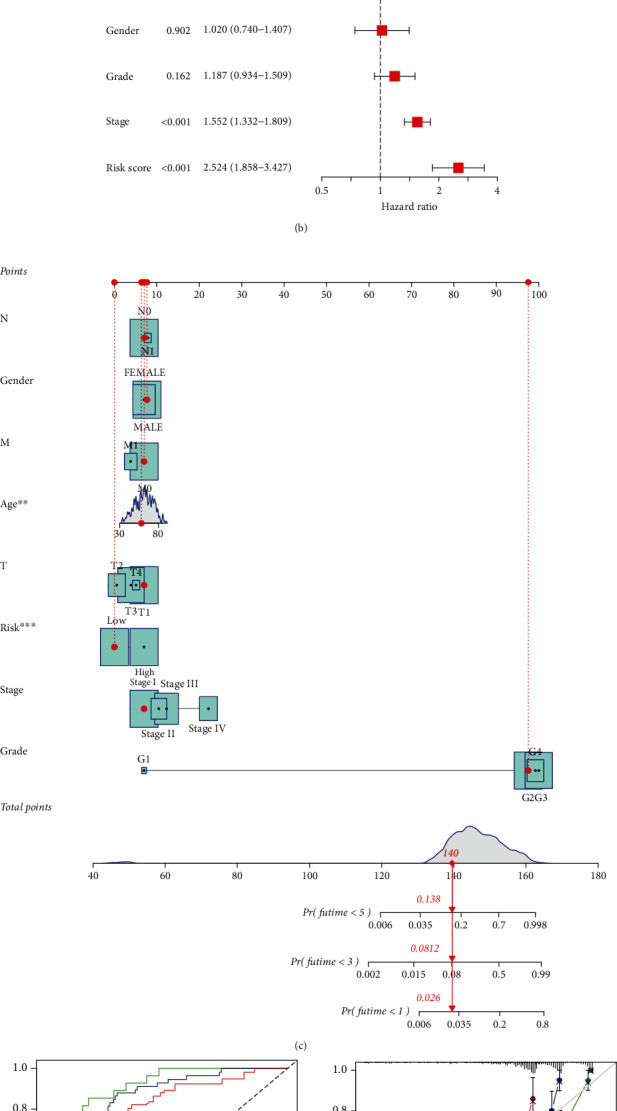
Univariate and multivariate Cox regression analyses and construction and analysis of nomogram. (a) Forest plot of univariate Cox regression analysis in TCGA-KIRC. (b) Forest plot of multivariate Cox regression analysis in TCGA-KIRC. (c) Nomogram constructed by combining based-IGPRM risk characteristic and clinical characteristics. (d) The 1-, 3-, and 5-year ROC and AUC predicted for the nomogram in the TCGA-KIRC cohort. (e) The calibration diagram of the nomogram for TCGA-KIRC cohort.

Since the risk score was substantially associated with the progression of KIRC, univariate and multivariate Cox regression analyses were performed to verify whether IGRPM-based risk characteristic could be used as an independent prognostic factor for KIRC patients. The results showed that the *p* value of the risk score was less than 0.001 in both univariate and multivariate regression analyses (Figures 7(a) and 7(b)), which confirmed that the risk score based on IGRPM was an independent variable affecting the survival of KIRC patients. Combining with the patient's age, sex, TNM staging, grade, and risk score, we designed a nomogram to expand clinical applicability of the risk characteristic based on IGRPM (Figure 7(c)). By scoring the comprehensive characteristics of each patient, in a certain range, the higher the total scored, the worse the prognosis of the patient would be. In addition, the ROC proved that nomogram could accurately judge the survival status of KIRC patients (Figure 7(d)). Moreover, the calibration diagram showed a good similarity between the performance of the nomogram and the ideal model (Figure 7(e)).

### 3.8. Validation of the Expression of Genes Used to Construct IGRPM in Early KIRC and Adjacent Normal Tissues

**Figure 8:**
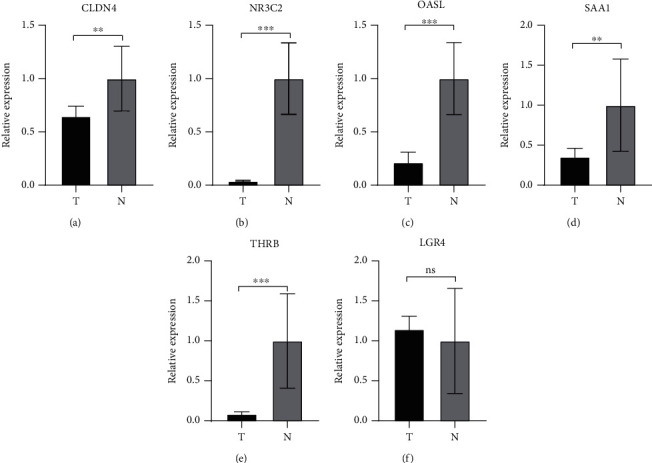
The expressions of some genes used to construct IGRPM in early KIRC and adjacent normal tissues by RT-qPCR. The relative expressions of CLDN4 (a), NR3C2 (b), OASL (c), SAA1 (d), THRB (e), and LGR4 (f) in early KIRC compared with adjacent normal tissues. T: KIRC tissues; N: adjacent normal tissues; ∗*p* < 0.05, ∗∗*p* < 0.01, and ∗∗∗*p* < 0.001; ns: the difference was no statistically significant.

**Figure 9:**
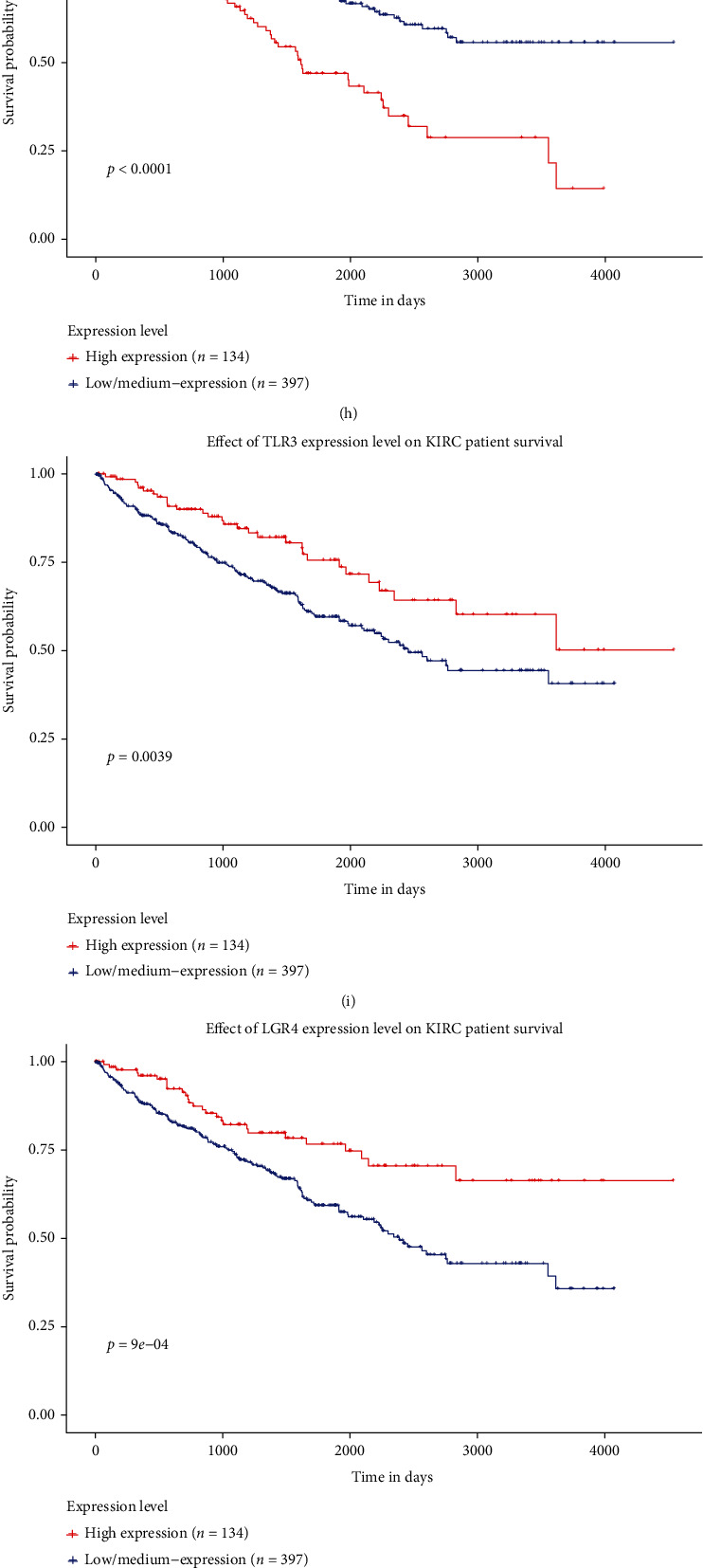
The expressions of genes used to construct IGRPM on the prognosis of KIRC. (a) OASL; (b) NR3C2; (c) THRB; (d) SAA1; (e) HNF4A; (f) SLC11A1; (g) SEMA6D; (h) CXCL5; (i) TLR3; (j) LGR4; (k) INHBE; (l) CSF1; (m) CLDN4; (n) PLA2G2A; (o) CRP; (p) TNFSF14; (q) PAEP.

In order to further deepen the understanding of the genes used to construct IGRPM, we verified the expressions of some of these genes in early KIRC and adjacent normal tissues. The results of RT-qPCR indicated that the expressions of CLDN4, NR3C2, OASL, SAA1, and THRB in early KIRC were considerably suppressed than those in adjacent normal tissues (Figures 8(a)–8(e)); however, the expression of LGR4 in early KIRC was no statistical significance compared with that in adjacent normal tissues (Figure 8(f)). These results demonstrated that not all genes used to construct IGRPM were significantly differentially expressed in early KIRC compared with adjacent normal tissues. Last but not least, we explored the impact of all genes used to construct IGRPM on the prognosis of KIRC through the UALCAN database. To our surprising, the expressions of all these genes significantly affected the OS of KIRC (Figures 9(a)–9(q)), which showed that all these genes used to construct IGRPM had a significant impact on the prognosis of KIRC, although not all these genes showed differential expression in early KIRC compared with adjacent normal tissues. Therefore, it was more accurate to judge the prognosis of KIRC patients by integrating the expressions of these genes, which indicated that the IGRPM constructed by us was also more reliable and meaningful than the expression of a single or few genes to judge the prognosis of KIRC patients.

## 4. Discussion

As the prognosis of KIRC patients varies greatly, it is particularly important to establish a stable classifier for risk stratification of different KIRC patients, which can maximize the individualized treatment and timely follow-up of KIRC patients. Therefore, scientists have invested a lot of time and energy to explore the complex mechanism of KIRC, but the current understanding of KIRC, especially the understanding of TIM and immune-related prognostic factors of KIRC, is far from satisfactory. In this study, the multifaceted mining of transcriptome data, tumor immune-related characteristics, and TMB was designed to comprehensively understand the immune characteristics of KIRC and to construct a simple and practical method to help clinicians determine the prognosis of patients with KIRC, so as to develop personalized treatment options for KIRC patients.

First of all, we divided KIRC into two immune-related subtypes, Immunity_L and Immunity_H, and analyzed the molecular characteristics of subtype specificity, such as immune-related gene expression, function, and pathway enrichment. Immunity_H had lower tumor purity and higher immune score than Immunity_L, indicating that immune cells exerted critical role in inhibiting tumor progression. These findings supported previous researches that the activation of immune-related responses can effectively control the progression of KIRC [28, 29]. Additionally, Immunity_H had higher levels of HLA genes expression and immune cells infiltration than Immunity_L, indicating that Immunity_H had stronger immunogenicity. It has been reported that the expressions of HLA genes play an important role in antitumor activity and in the recognition and elimination of cancer cells [30, 31]. Then we visualized the DEGs and DEIGs in Immunity_H and Immunity_L, and it could be seen that the expressions of immune-related genes were found to be significantly different in the two groups. GO and KEGG analyses showed that Immunity_H had more abundant immune-related signals than Immunity_L. These results demonstrated the accuracy and necessity of dividing KIRC into two subtypes associated with immunity. Then we combined the gene expression data and survival data of TCGA and GEO to identify the genes that may have a significant impact on the prognosis of KIRC patients. We screened out 47 genes that had a significant impact on the prognosis of KIRC patients. Most of these genes have been reported in previous studies whose results were similar to ours. For example, it has been reported that NR3C2 expression could be employed as an independent prognostic factor of KIRC [32], and overexpression of NR3C2 have potential to restrict the proliferation and invasion of KIRC [33]. For HNF4A (Hepatocyte Nuclear Factor 4, Alpha), a tumor suppressor, its expression could affect the prognosis of KIRC [34]. Overexpression of IKBKE (Inhibitor of Kappa Light Polypeptide Gene Enhancer in B-cells, Kinase Epsilon) is associated with an increased risk of death in KIRC [35]. These results fully showed that the PIGs screened out by us had great reliability and that our method of screening prognostic genes had certain superiority. Then coexpression analysis of KIRC-related transcription factors and PIGs was performed, and their interactions were again demonstrated by PPI. It has been reported that PPARG (peroxisome proliferation-activated receptor gamma) promotes apoptosis and inhibits the migration and proliferation of KIRC by inhibiting SIX2 (SIX homeobox 2) [36]. BATF (Basic Leucine Zipper ATF-Like Transcription Factor) affects the prognosis of many kinds of cancer by combining with HHLA2 (HERV-H LTR-associating 2) DNA, especially the prognosis of KIRC [37]. CIITA (Class II Major Histocompatibility Complex Transactivator) methylation is associated with immune heterogeneity in hematological tumors [38]. These transcription factors are associated with tumors through different mechanisms. All these results provided a basis for us to further understand the occurrence and development mechanism of KIRC. Based on the above information, we could have a more comprehensive understanding of the relationship between KIRC and immunity, and dividing KIRC patients into different subtypes through immune-related genes would lay a foundation for us to estimate the prognosis of KIRC patients from a new perspective.

Based on the expressions of 17 immune genes, we constructed an IGRPM to assess the prognosis of patients with KIRC and evaluated the reliability of the model through ROC and calibration diagram. The results demonstrated that the risk score was negatively correlated with the OS of KIRC patients, and there was a substantial difference in survival time between the high-risk score group and low-risk score group. Moreover, most of the 17 genes used to construct IGRPM are related to the onset and progression of cancer and have the potential to become therapeutic targets for cancer, such as OASL [39], NR3C2 [32, 33], SAA1 [40], CXCL5 (C-X-C Motif Chemokine Ligand 5) [41], TLR3(toll-like receptor 3) [42], CLDN4 [43, 44], THRB [45], and LGR4 [46]. However, the specific molecular mechanisms of these genes affecting the prognosis of tumor patients are not clear, so the molecular mechanisms of the effect of these genes on KIRC patients need to be further studied in the future. Nevertheless, we speculated that the 17 genes affecting the prognosis of KIRC patients were closely related to the TIM, because these 17 genes were significantly associated with 22 types of human immune cells.

For the sake of further confirming the scientificity of classifying KIRC patients into the high- and low-risk groups, we compared the TMB of the two groups and found that the TMB of the high-risk group was substantially elevated compared with that of the low-risk group, and the survival time of KIRC patients with L-TMB was significantly longer than that of KIRC patients with H-TMB. This was consistent with the conclusion that TMB could be used as a biomarker to judge the prognosis of tumor patients reported in other researches [47, 48]. Similar results have been obtained from the research of lung squamous cell carcinoma [49]. Moreover, combining the risk score and TMB, we found that L-TMB + low-risk was most beneficial to the survival outcome of patients with KIRC, which provided a more accurate method for us to evaluate the prognosis of KIRC patients.

Finally, through univariate and multivariate Cox regression analyses, we proved that the risk characteristic based on IGRPM could be employed as an independent prognostic factor for patients with KIRC. Meanwhile, in order to make it convenient for clinicians to judge the prognosis of KIRC patients simply and effectively, we constructed a nomogram combining the clinicopathological information and the risk score of patients and verified its reliability by ROC and calibration diagram. Last but not the least, we verified the expressions of some of these genes used to construct IGRPM in early KIRC and adjacent normal tissue by RT-qPCR. The RT-qPCR results demonstrated that not all these genes used to construct IGRPM were significantly differentially expressed in early KIRC compared with adjacent normal tissue, but all these genes had significant influence on the prognosis of KIRC. Because of this, it further indicated that it was more accurate and effective to judge the prognosis of patients with KIRC by risk stratification based on the expression of these genes than by the expression of a single or few genes.

Although our study could make an important contribution to differentiating the immune status and predicting the prognosis of patients with KIRC, admittedly, there are some limitations to our study. On the one hand, the immune-related features of KIRC are constructed through public datasets, but these features have not yet been verified by clinical cohorts. On the other hand, due to the different information contained in TCGA and GEO databases, some data can only be obtained from TCGA, which makes some of the data less representative. Nevertheless, our results still have important guiding significance for survival prediction and immune assessment of patients with KIRC and may promote the development of immunotherapy for KIRC.

## 5. Conclusion

In a word, we elucidated the information of the interaction between tumor and immunity in KIRC from different aspects and used a variety of algorithms to establish IGRPM-based risk characteristic to evaluate the prognosis of KIRC patients. In addition, we constructed a nomogram which was convenient for clinicians to combine the clinicopathological information of patients to judge the prognosis of patients with KIRC. These comprehensive immune assessments and survival predictions, integrating multiple aspects of data and clinical information, can provide additional value to the current Tumor Node Metastasis staging system for risk stratification of KIRC and may facilitate the development of KIRC immunotherapy.

## Data Availability

The data used to support the findings of this study are available in TCGA (https://gdc.cancer.gov/) and GEO databases. The raw data of RT-qPCR will be made available by the authors, without undue reservation, to any qualified researcher.

## Supplementary Materials

Supplementary Materials table1: the characteristics of patients for RT-qPCR. Supplementary table 2: the coexpression relationship between PIGs and DETFs. Supplementary table 3: the gene and coef used to calculate the risk score for each sample. Supplementary file1: the corrected gene expression level, survival time, and survival status of TCGA and GEO samples.
