# Supplementary material for: Identification of Survival Risk and Immune-Related Characteristics of Kidney Renal Clear Cell Carcinoma
Source: J Immunol Res. 2022 Jul 4;2022:6149369. doi: 10.1155/2022/6149369 (PMC9273399; doi:10.1155/2022/6149369)
Supplement: Supplementary Materials — table1: the characteristics of patients for RT-qPCR. Supplementary table 2: the coexpression relationship between PIGs and DETFs. Supplementary table 3: the gene and coef used to calculate the risk score for each sample. Supplementary file1: the corrected gene expression level, survival time, and survival status of TCGA and GEO samples. [file 6149369.f1.zip › Supplementary table1.docx]

**Supplementary table1:**

The characteristics of patients for RT-qPCR

| Patients | Sex | Years of age | Tumor location | Tumor size (cm) | TNM stage | Histological type |
| --- | --- | --- | --- | --- | --- | --- |
| Patient1 | Male | 47 | Right | 4.0x3.0x2.5 | T_1_N_0_M_0_ | KIRC |
| Patient2 | Female | 52 | Right | 3.5x3.0x3.0 | T_1_N_0_M_0_ | KIRC |
| Patient3 | Male | 55 | Right | 3.0x2.5x2.0 | T_1_N_0_M_0_ | KIRC |
