# Supplementary material for: Identification of Survival Risk and Immune-Related Characteristics of Kidney Renal Clear Cell Carcinoma
Source: J Immunol Res. 2022 Jul 4;2022:6149369. doi: 10.1155/2022/6149369 (PMC9273399; doi:10.1155/2022/6149369)
Supplement: Supplementary Materials — table1: the characteristics of patients for RT-qPCR. Supplementary table 2: the coexpression relationship between PIGs and DETFs. Supplementary table 3: the gene and coef used to calculate the risk score for each sample. Supplementary file1: the corrected gene expression level, survival time, and survival status of TCGA and GEO samples. [file 6149369.f1.zip › Supplementary table2.docx]

**Supplementary Table 2：**

The co-expression relationship between PIGs and DETFs

| PIGs | DETFs | cor | pvalue | FDR |
| --- | --- | --- | --- | --- |
| OASL | FOXP3 | 0.42768 | 2.22E-25 | 2.03E-24 |
| OASL | IRF1 | 0.446563 | 8.87E-28 | 8.84E-27 |
| OASL | BATF | 0.481886 | 1.09E-32 | 1.47E-31 |
| OASL | EOMES | 0.404322 | 1.29E-22 | 1.07E-21 |
| TNFSF13B | STAT4 | 0.497373 | 4.99E-35 | 7.51E-34 |
| TNFSF13B | CEBPA | 0.434489 | 3.16E-26 | 2.99E-25 |
| TNFSF13B | FOXP3 | 0.495802 | 8.73E-35 | 1.29E-33 |
| TNFSF13B | IRF1 | 0.464885 | 2.97E-30 | 3.38E-29 |
| TNFSF13B | BATF | 0.620234 | 1.30E-58 | 5.19E-57 |
| TNFSF13B | CIITA | 0.459813 | 1.49E-29 | 1.60E-28 |
| TNFSF13B | STAT1 | 0.690202 | 1.79E-77 | 1.43E-75 |
| TNFSF13B | IKZF1 | 0.722121 | 5.44E-88 | 7.23E-86 |
| TNFSF13B | EOMES | 0.568434 | 1.88E-47 | 4.82E-46 |
| NR3C2 | GATA2 | 0.598069 | 1.38E-53 | 4.57E-52 |
| NR3C2 | PPARG | 0.561606 | 3.98E-46 | 9.34E-45 |
| NR3C2 | SREBF2 | 0.684244 | 1.16E-75 | 8.44E-74 |
| NR3C2 | FOXP1 | 0.677833 | 9.32E-74 | 6.19E-72 |
| NR3C2 | FOXO1 | 0.731372 | 2.55E-91 | 4.07E-89 |
| THRB | GATA2 | 0.508247 | 9.60E-37 | 1.66E-35 |
| THRB | PPARG | 0.574634 | 1.10E-48 | 3.02E-47 |
| THRB | SREBF2 | 0.637262 | 9.39E-63 | 4.16E-61 |
| THRB | FOXP1 | 0.669578 | 2.24E-71 | 1.37E-69 |
| THRB | FOXO1 | 0.69965 | 1.93E-80 | 1.92E-78 |
| LILRB3 | STAT4 | 0.486349 | 2.38E-33 | 3.32E-32 |
| LILRB3 | FOXP3 | 0.478241 | 3.73E-32 | 4.87E-31 |
| LILRB3 | BATF | 0.571846 | 3.97E-48 | 1.05E-46 |
| LILRB3 | CIITA | 0.511864 | 2.50E-37 | 4.43E-36 |
| LILRB3 | IKZF1 | 0.480562 | 1.71E-32 | 2.27E-31 |
| LILRB3 | EOMES | 0.414257 | 9.16E-24 | 7.93E-23 |
| IL6 | EPO | 0.424263 | 5.82E-25 | 5.27E-24 |
| KITLG | FOXP1 | 0.476778 | 6.08E-32 | 7.82E-31 |
| KITLG | FOXO1 | 0.499838 | 2.06E-35 | 3.16E-34 |
| CD72 | STAT4 | 0.567195 | 3.28E-47 | 8.18E-46 |
| CD72 | CEBPA | 0.462789 | 5.80E-30 | 6.42E-29 |
| CD72 | FOXP3 | 0.531444 | 1.28E-40 | 2.76E-39 |
| CD72 | IRF1 | 0.647144 | 2.80E-65 | 1.49E-63 |
| CD72 | BATF | 0.786098 | 2.75E-114 | 1.10E-111 |
| CD72 | CIITA | 0.618842 | 2.77E-58 | 1.05E-56 |
| CD72 | STAT1 | 0.657128 | 6.28E-68 | 3.58E-66 |
| CD72 | IKZF1 | 0.74052 | 9.46E-95 | 1.88E-92 |
| CD72 | EOMES | 0.740677 | 8.23E-95 | 1.88E-92 |
| PPARG | FOXO1 | 0.458745 | 2.08E-29 | 2.22E-28 |
| ISG20 | STAT4 | 0.504697 | 3.54E-36 | 5.76E-35 |
| ISG20 | FOXP3 | 0.526942 | 7.65E-40 | 1.56E-38 |
| ISG20 | BATF | 0.644851 | 1.10E-64 | 5.48E-63 |
| RNASE2 | STAT1 | 0.421438 | 1.28E-24 | 1.15E-23 |
| RNASE2 | IKZF1 | 0.429379 | 1.37E-25 | 1.27E-24 |
| TNFRSF18 | STAT4 | 0.466711 | 1.65E-30 | 1.91E-29 |
| TNFRSF18 | FOXP3 | 0.583478 | 1.73E-50 | 5.10E-49 |
| TNFRSF18 | BATF | 0.608272 | 7.50E-56 | 2.60E-54 |
| LGR4 | SREBF2 | 0.400565 | 3.44E-22 | 2.77E-21 |
| LGR4 | FOXO1 | 0.471828 | 3.13E-31 | 3.72E-30 |
| CSF1 | FOXP3 | 0.446271 | 9.69E-28 | 9.54E-27 |
| CSF1 | IRF1 | 0.41884 | 2.62E-24 | 2.30E-23 |
| CSF1 | BATF | 0.468109 | 1.05E-30 | 1.23E-29 |
| CSF1 | IKZF1 | 0.447459 | 6.77E-28 | 6.92E-27 |
| XCL1 | STAT4 | 0.512801 | 1.76E-37 | 3.19E-36 |
| XCL1 | CEBPA | 0.409148 | 3.61E-23 | 3.06E-22 |
| XCL1 | FOXP3 | 0.60852 | 6.59E-56 | 2.39E-54 |
| XCL1 | IRF1 | 0.49483 | 1.23E-34 | 1.79E-33 |
| XCL1 | BATF | 0.703801 | 8.80E-82 | 1.00E-79 |
| XCL1 | IKZF1 | 0.475714 | 8.67E-32 | 1.09E-30 |
| XCL1 | EOMES | 0.563126 | 2.03E-46 | 4.90E-45 |
| ESRRG | FOXO1 | 0.488164 | 1.27E-33 | 1.81E-32 |
| IL15RA | STAT4 | 0.513273 | 1.47E-37 | 2.73E-36 |
| IL15RA | FOXP3 | 0.519067 | 1.63E-38 | 3.17E-37 |
| IL15RA | IRF1 | 0.520293 | 1.02E-38 | 2.03E-37 |
| IL15RA | BATF | 0.642916 | 3.46E-64 | 1.62E-62 |
| IL15RA | IKZF1 | 0.401106 | 2.99E-22 | 2.43E-21 |
| IL15RA | EOMES | 0.402283 | 2.20E-22 | 1.81E-21 |
| IKBKE | STAT4 | 0.407654 | 5.37E-23 | 4.51E-22 |
| IKBKE | CEBPA | 0.432946 | 4.93E-26 | 4.62E-25 |
| IKBKE | FOXP3 | 0.528039 | 4.97E-40 | 1.04E-38 |
| IKBKE | BATF | 0.627059 | 3.06E-60 | 1.28E-58 |
| IKBKE | STAT1 | 0.475695 | 8.72E-32 | 1.09E-30 |
| IKBKE | IKZF1 | 0.473123 | 2.04E-31 | 2.47E-30 |
| IKBKE | EOMES | 0.438083 | 1.11E-26 | 1.06E-25 |
| CXCL13 | STAT4 | 0.485193 | 3.54E-33 | 4.86E-32 |
| CXCL13 | FOXP3 | 0.505654 | 2.50E-36 | 4.14E-35 |
| CXCL13 | IRF1 | 0.438403 | 1.01E-26 | 9.79E-26 |
| CXCL13 | BATF | 0.691145 | 9.16E-78 | 8.12E-76 |
| CXCL13 | STAT1 | 0.457764 | 2.84E-29 | 2.97E-28 |
| CXCL13 | IKZF1 | 0.503199 | 6.12E-36 | 9.56E-35 |
| CXCL13 | EOMES | 0.47322 | 1.98E-31 | 2.43E-30 |
| HCST | STAT4 | 0.461233 | 9.51E-30 | 1.04E-28 |
| HCST | CEBPA | 0.451276 | 2.12E-28 | 2.19E-27 |
| HCST | FOXP3 | 0.555798 | 5.07E-45 | 1.15E-43 |
| HCST | IRF1 | 0.507984 | 1.06E-36 | 1.79E-35 |
| HCST | BATF | 0.837959 | 2.35E-143 | 1.87E-140 |
| HCST | IKZF1 | 0.447143 | 7.45E-28 | 7.51E-27 |
| HCST | EOMES | 0.503606 | 5.27E-36 | 8.41E-35 |
| TNFSF4 | STAT4 | 0.464783 | 3.07E-30 | 3.45E-29 |
| TNFSF4 | FOXP3 | 0.420596 | 1.61E-24 | 1.43E-23 |
| TNFSF4 | IRF1 | 0.516155 | 4.96E-38 | 9.40E-37 |
| TNFSF4 | BATF | 0.538602 | 7.10E-42 | 1.57E-40 |
| TNFSF4 | CIITA | 0.41317 | 1.23E-23 | 1.05E-22 |
| TNFSF4 | STAT1 | 0.589639 | 8.88E-52 | 2.72E-50 |
| TNFSF4 | IKZF1 | 0.576972 | 3.71E-49 | 1.06E-47 |
| TNFSF4 | EOMES | 0.591673 | 3.29E-52 | 1.05E-50 |
