# Supplementary material for: Identification of Survival Risk and Immune-Related Characteristics of Kidney Renal Clear Cell Carcinoma
Source: J Immunol Res. 2022 Jul 4;2022:6149369. doi: 10.1155/2022/6149369 (PMC9273399; doi:10.1155/2022/6149369)
Supplement: Supplementary Materials — table1: the characteristics of patients for RT-qPCR. Supplementary table 2: the coexpression relationship between PIGs and DETFs. Supplementary table 3: the gene and coef used to calculate the risk score for each sample. Supplementary file1: the corrected gene expression level, survival time, and survival status of TCGA and GEO samples. [file 6149369.f1.zip › Supplementary table3.docx]

**Supplementary table 3：**

The gene and coef used to calculate the risk score for each sample

| Gene | Coef |
| --- | --- |
| OASL | 0.09585 |
| NR3C2 | -0.00135 |
| THRB | -0.22465 |
| SAA1 | 0.031116 |
| HNF4A | -0.12009 |
| SLC11A1 | 0.15524 |
| SEMA6D | -0.02817 |
| CXCL5 | 0.076239 |
| TLR3 | -0.05471 |
| LGR4 | -0.04426 |
| INHBE | 0.032537 |
| CSF1 | 0.009111 |
| CLDN4 | -0.14756 |
| PLA2G2A | 0.084453 |
| CRP | 0.017257 |
| TNFSF14 | 0.042309 |
| PAEP | 0.020268 |
